# Antinociceptive Effects of Sinomenine Combined With Ligustrazine or Paracetamol in Animal Models of Incisional and Inflammatory Pain

**DOI:** 10.3389/fphys.2020.523769

**Published:** 2021-02-09

**Authors:** Tianle Gao, Tao Li, Wei Jiang, Weiming Fan, Xiao-Jun Xu, Xiaoliang Zhao, Zhenming Yin, Huihui Guo, Lulu Wang, Jun Gao, Yanxing Han, Jian-Dong Jiang, Danqiao Wang

**Affiliations:** ^1^State Key Laboratory of Bioactive Substances and Function of Natural Medicine, Institute of Materia Medica, Chinese Academy of Medical Sciences, Beijing, China; ^2^Beijing Key Laboratory of Traditional Chinese Medicine Basic Research on Prevention and Treatment of Major Diseases, Experimental Research Center, China Academy of Chinese Medical Sciences, Beijing, China; ^3^Zhejiang Zhenyuan Pharmaceutical Co., Ltd, Shaoxing, China; ^4^Department of Physiology and Pharmacology, Karolinska Institutet, Stockholm, Sweden; ^5^Department of Neurosurgery, Peking Union Medical College Hospital, Chinese Academy of Medicine Sciences & Peking Union Medical College, Beijing, China

**Keywords:** neuroimmune interaction, post-operative pain, carrageenan-induced inflammation, drug combinations, pharmacokinetics, microdialysis

## Abstract

The management of postoperative and inflammatory pain has been a pressing challenge in clinical settings. Sinomenine (SN) is a morphinan derived alkaloid with remarkable analgesic properties in various kinds of pain models. The aim of the current study is to investigate if SN can enhance the effect of ligustrazine hydrochloride (LGZ) or paracetamol (PCM) in animal models of postoperative and inflammatory pain. And to determine if the combined therapeutic efficacies can be explained by pharmacokinetics changes. Pharmacological studies were performed using a rat model of incisional pain, and a mouse model of carrageenan induced inflammatory pain. Pharmacokinetic studies were performed using a microdialysis sampling and HPLC-MS/MS assay method to quantify SN, LGZ, and PCM levels in blood and extracellular fluid in brain. We found that SN plus LGZ or SN plus PCM produced marked synergistic analgesic effects. However, such synergy was subjected to pain modalities, and differed among pain models. Pharmacological discoveries could be partially linked to pharmacokinetic alterations in SN combinations. Though further evaluation is needed, our findings advocate the potential benefits of SN plus LGZ for postoperative pain management, and SN plus PCM for controlling inflammatory pain.

## Introduction

Pain generated from operation or inflammation is an important clinical concern. However, current medication is far from satisfactory. Less than half of patients report adequate postoperative pain relief (Chou et al., 2016) following operation, and up to 60% of patients with inflammatory rheumatic diseases experience persistent pain left to be treated (Häuser and Fitzcharles, 2017). Undermanaged pain prevents recovery and induces physical and mental impairments (Breivik et al., 2006; Langley, 2011). Moreover, when left untreated, the acute pain may undergo a “chronification” process to produce treatment-resistant pain, leading to a tremendous reduction in quality of life (Breivik et al., 2006). Therefore, finding effective analgesic methods has become a pressing challenge.

Traditional Chinese medicine has a long history with rich clinical experience in pain therapy. We turned to this natural resource to search for novel analgesics and discovered sinomenine (SN) as a candidate. SN is a morphinan derived alkaloid purified from the root of the climbing plant *Sinomenium Acutum*. It was traditionally used as herbal medicine for rheumatism and arthritis (Feng et al., 2007; Xu et al., 2008). Clinical studies demonstrated that pain in many types of neuralgia can be relieved by SN (Yamasaki, 1976), suggesting a dual therapeutic role of SN on inflammation and pain. In previous studies, we have demonstrated that SN can alleviate acute pain, neuropathic pain and chronic inflammatory pain arising from rheumatoid arthritis, without introducing tolerance or observable side effects (Gao et al., 2013, 2014, 2015).

During incision or inflammation, a broad range of inflammatory mediators forming an “inflammatory soup” (Boddeke, 2001; Mantyh et al., 2002), and increasing the responsiveness of sensory neurons (Scholz and Woolf, 2002; Woolf and Ma, 2007). As SN has distinct immunoregulative property (Jiang et al., 2020), it may alter the unbalanced neuroimmune interaction and decrease the hyperresponsiveness of sensory neurons in incisional or inflammatory pain states. Indeed, supporting evidences revealed that SN was effective in reducing carrageenan induced inflammatory pain in mice (Gao et al., 2013), and incisional pain in rats (Zhu et al., 2016).

Combing drugs with different mechanisms of actions is usually a beneficial attempt in clinical pain management. Advantage of such combination therapies includes improved analgesia and / or reduced dosages of individual drugs, thus minimizing side effects (Vorobeychik et al., 2011). Recently, we have shown that SN could enhance the analgesic potency of ligustrazine (LGZ, also known as Tetramethylpyrazine, the active substance extracted from plant *Ligusticum chuanxiong Hort*), which is effective on acute inflammatory pain (Liang et al., 2004), and pain after traumatic injury (Gao et al., 2019a). Since SN, LGZ and the widely used painkiller paracetamol (PCM, also known as acetaminophen) are promising non-opioid analgesics. It is worth to determine if SN could generate synergistic interactions with LGZ or PCM against pain induced by incision or inflammation.

Hence, in the present study, we evaluated the antinociceptive effects of SN, LGZ, and PCM from sub-effective to effective levels, as well as examined the pharmacological interactions between SN and LGZ or PCM, using animal models of incisional pain and carrageenan induced inflammatory pain. In addition, we measured pharmacokinetics of SN, LGZ, and PCM in single and combined settings, so that pharmacokinetic and pharmacological studies could be correlated. The aim of this article is to validate: (1) If SN combined with LGZ or PCM could achieve synergy; (2) If efficacies of combinational therapies could be differed between inflammatory pain and incisional pain conditions; and (3) If the pharmacokinetic properties of individual drug can be influenced by combinational formulas.

## Materials and Methods

### Animals

The animals used were Sprague-Dawley rats, male, weighing 250–300 g (from Harlan, The Netherlands; or Beijing Vital River Laboratory Animal Technology, China), or C57BL/6 mice, male, weighing 20–30 g (from Charles River, Sweden, or Beijing Vital River Laboratory Animal Technology, China). Animals were housed 4 per cage for rats or 5 per cage for mice, at a constant room temperature of 22°C in a 12:12 h light-dark cycle with *ad libitum* access to food and water.

### Rat Model of Incisional Pain

We used a rat model of incisional pain that has been reported previously (Brennan et al., 1996). In brief, rats were anesthetized with Domitor (75 mg/kg ketamine + 1 mg/kg medetomidine in 1 ml/kg), and a 1 cm longitudinal incision was made through the skin, fascia and muscle of the plantar aspect of the hind paw. The surgery wound was covered with an antibiotic ointment (Bacitracin Zinc Ointment, Actavis Pharma, Inc., USA), to prevent infections. Mechanical and heat hypersensitivities in the hind paw with incision reached peak value around 24 h after surgery.

For measurement of mechanical hypersensitivity. The rats were placed in plastic cages with a metal mesh floor. After habituation for 1 h, the plantar surface of the hind paw adjacent to the skin incision was stimulated with a set of calibrated von Fray hairs (Stoelting, USA) with increasing force. Each filament was applied 5 times and response threshold was reached when the animal withdrew the paw at least 3 times. The cut-off value was 60 g. Heat hyperalgesia was measured by focusing a radiant heat source (Ugo Basile, Italy) on the plantar surface of hind paw adjacent to the incision. The hind paw withdrawal latency was automatically recorded. The intensity of the stimulation was adjusted and fixed so that the baseline latency for normal animals were around 2 to 6 s and the cut-off value was set at 20 s.

### Mouse Model of Carrageenan Induced Inflammatory Pain

As reported in our earlier studies (Hao et al., 1998; Gao et al., 2013), mice were anesthetized with 75 mg/kg ketamine + 1 mg/kg medetomidine, in a volume of 1 ml/kg. λ-carrageenan (20 μl, 2%, Sigma-Aldrich, Germany) was injected subcutaneously (s.c.) into the plantar surface of one hind paw. Mechanical and heat hypersensitivities of the inflamed hind paw was peaked 24 h after the injection.

The paw-withdrawal threshold to mechanical stimulation in mice was tested using a set of calibrated von Fray hairs (Stoelting, USA), with the cut-off value set at 4 g. For test of heat hyperalgesia, mice were gently restrained and a radiant heat source (Hargreaves' test, Ugo Basile, Italy) was focused on the plantar surface of hind paw. The hind paw withdrawal latency was automatically recorded. The intensity of the stimulation was adjusted and fixed so that the baseline latency for normal mice were within 2 to 6 s and the cut-off value was set at 15 s.

### Pharmacological Study Design

In this study, behavioral studies were performed under intraperitoneal (i.p.) administrations to be consistent (and can be correlated) with our earlier published results (Gao et al., 2013, 2019a). The timeline of pharmacological interventions was illustrated in the Supplementary Figure 1. SN/Vehicle was given 60 min prior to the application of LGZ/PCM/Vehicle. Pain behavioral measurement (measurement of mechanical allodynia and heat hyperalgesia) was performed after application of LGZ/PCM/Vehicle, for 240 min. This pharmacological intervention strategy was inherited from our previous study (Gao et al., 2019a), in which we show that the analgesic efficacy of SN combination is optimal when SN was given 60 min prior to the combinational drug. To assess the analgesic effects of SN combinations in incisional pain and inflammatory pain models, animals were randomly assigned to following treatment groups (6–8 rats/mice per group): SN low dose group (SN 10 mg/kg); SN middle dose group (SN 20 mg mg/kg); SN high dose group (SN 80 mg/kg); LGZ low dose group (LGZ 10 mg/kg); LGZ middle dose group (LGZ 20 mg/kg); LGZ high dose group (LGZ 80 mg/kg); SN+LGZ low dose group (SN 10 mg/kg + LGZ 10 mg/kg); SN+LGZ middle dose group (SN 20 mg/kg + LGZ 10 mg/kg); SN+LGZ high dose group (SN 20 mg/kg + LGZ 20 mg/kg); PCM low dose group (PCM 10 mg/kg); PCM middle dose group (PCM30 mg/kg); PCM high dose group (PCM 100 mg/kg); SN+PCM low dose group (SN 10 mg/kg + PCM 10 mg/kg); SN+PCM middle dose group (SN 20 mg/kg + PCM 10 mg/kg); SN+PCM high dose group (SN 20 mg/kg + PCM 30 mg/kg); and Vehicle groups (for all separate experiments).

### Pharmacokinetic Study Design

Pharmacokinetic studies are performed intravenously (i.v.), to avoid deviations in distribution time (of each drug, form i.p. injection site to blood circulation), so that we can investigate the potential drug-drug interactions in a stable manner. Rats were randomly assigned to 7 treatment groups (6 rats per group): SN group (SN50 mg/kg); LGZ group (LGZ50 mg/kg); PCM group (PCM50 mg/kg); SN+LGZ high dose group (SN50 mg/kg + LGZ 50 mg/kg); SN+LGZ low dose group (SN 25 mg/kg + LGZ 25 mg/kg); SN+PCM high dose group (SN 50 mg/kg + PCM 50 mg/kg); SN+PCM low dose group (SN 25 mg/kg + PCM 25 mg/kg). In each group, an injection catheter (C19PU, Instech Laboratories, USA) was implanted into the left femoral vein of each rat, 1 day before i.v. drug administration. The i.v. drug administration volume was 0.5 mL/100 g (of rat body weight), and injections were completed within 1 min.

### Blood and Brain Microdialysis Surgery and Microdialysate Sampling

After anaesthetization, the blood microdialysis probe (4 mm-effective membrane length and 20 000 molecular weight cut-off, CMA Microdialysis, Sweden) was positioned within the jugular vein toward the right atrium and then perfused with anti-coagulant citrate dextrose (ACD) solution (Sigma-Aldrich, Germany) consisting of citric acid 3.5 mM, sodium citrate 7.5 mM, and dextrose 13.6 mM. The brain microdialysis probe (4 mm-effective membrane length and 20 000 molecular weight cut-off, CMA Microdialysis, Sweden) was implanted in the corpus striatum zone (AP +0.2 mm, ML 3 mm, and DV 3.5 mm) according to the atlas of rat brain (Paxinos and Watson, 1998), and perfused with Ringer's solution (consisting of NaCl 145.3 mM; KCl 4.01 mM; CaCl2 2.97 mM; pH 7.0). During the period of surgery, the body temperature of each rat was maintained at 37°C using a heating pad. Rats were kept in free-moving condition for 1 h prior to the initiation of sample collection for reaching equilibrium. After blank control dialysate samples were collected, animals received corresponding drugs followed by a 6 h dialysate sampling. The dialysates were collected at 20 min intervals at a perfusion rate of 1.5 μL min^−1^ using a CMA120 awake and freely moving system (CMA Microdialysis, Sweden). For all samples, a 10 min delay was added into the sampling procedure to compensate for the dead volume between the active membrane and the sample collection outlet.

### Microdialysis Probe Recovery

The concentrations of SN, LGZ, and PCM in blood and brain extracellular fluid (bECF) were calculated from the corresponding dialysate sample according to the following equation:

$$C_{blood}\text{or}C_{bECF}\;=\;C_{\text{d}}/R_{in\;vivo}\;=\;({\text{C}}_{\text{d}}{\text{/R}}_{\text{in vitro}})\times ({\text{D}}_{\text{in vitro}}{\text{/D}}_{\text{in vivo}})$$

where *C*_blood_ or *C*_bECF_ was the measured concentration of each drug in blood or bECF; *C*_d_ was the measured concentration of each drug in the dialysate sample; *R*_*in vivo*_ was the recovery of the probe inserted into the rat's blood or brain; *R*_*in vitro*_ was the recovery of the probe immersed in ACD or Ringer's solution containing drug and perfused with drug-free solution; *D*_*in vitro*_ was the delivery of drug calculated by measuring the loss in the compound concentration between the perfusate and the dialysate when the probe was immersed in drug-free solution and perfused with ACD or Ringer's solution containing drug; *D*_*in vivo*_ was the delivery of drug determined in the same way as *D*_*in vitro*_ except that the probe was inserted into the rat blood or brain. In the current study, the *R*_*in vivo*_ of SN, LGZ, and PCM in blood was 21.31, 20.61, and 24.73%, respectively. The *R*_*in vivo*_ of SN, LGZ and PCM in brain was 19.38, 20.11, and 25.33%, respectively.

### HPLC-MS/MS Quantification

The quantitative analyses were carried out using the method described earlier (Zhao et al., 2015; Gao et al., 2019b; Li et al., 2019, 2020), in which a HPLC-MS/MS system (AB Sciex LLC, USA) was applied. Reversed-phase separation was performed on an XSelect^@^ HSS T3 column (2.5 μm, 2.1×50 mm, Waters Corp., USA). The mobile phase consisted of (A) water/formic acid (100/0.005, v/v) and (B) methanol/ formic acid (100/0.005, v/v). Gradient elution was carried out for 6.0 min at a flow rate of 0.3 mL/min. Gradient conditions were as follows: 0-1.5 min, 15% B; 1.5-3.0 min, 15% B–100% B; 3.0–4.5 min, 100% B; 4.50–4.51 min, 100% B–15% B; 4.51–6.0 min, 15% B. One microliter aliquot of each sample was injected into the column. The column temperature was kept at 30°C. All samples were kept at 6°C throughout the analysis. Mass spectrometry was performed on an Sciex 6500+ triple quadrupole mass spectrometer equipped with a Turbo V ion source. All compounds were detected in positive electrospray ion mode. Curtain gas (CUR), nebulizer gas (GS1), and turbo-gas (GS2) were set at 20, 55, and 55 psi, respectively. The electrospray voltage was +5.5 kV, and the turbo-ion spray source temperature was 550°C. Nitrogen was employed as the collision gas. The system was operated in multiple reaction monitoring (MRM) mode. Product ion spectrums and fragmentation pattern of SN, LGZ, PCM and naloxone (internal standard) were illustrated in Supplementary Figure 2. The precursors and product ions of LGZ, PCM, and SN were 137/80 (CE: 41; DP: 74), 152/110 (CE: 24; DP: 30), and 330/181 (CE: 44; DP: 158), respectively. Ions and fragmentations used in MRM mode for each compound were illustrated in Supplementary Table 1. Representative extraction ion chromatograms and retention time of each compound were illustrated in Supplementary Figure 3. Data acquisitions were performed using Analyst 1.7 software (Applied Biosystems, USA). Multiquant software (Applied Biosystems, USA) was used to quantify all drugs.

### Pharmacokinetic Data Analysis

The blood and bECF pharmacokinetic parameters were estimated by a non-compartmental method using the DAS software package (version 3.0, BioGuider Co., Shanghai, China). The maximal concentration (Cmax) and time taken to achieve peak concentration (Tpeak) were observed values with no interpolation. The area under the concentration–time curve up to 6 h (AUC0→ 6 h) was calculated using trapezoidal rule. The t_1/2_ was calculated using the relationship of 0.693/k, where k was the constant elimination rate. The clearance (CL) for i.v. dosing was calculated as dividing the administered dosage by the AUC 0→ 6 h. The distribution volume (Vss) for i.v. dosing was calculated as multiplying the CL by the mean residence time (MRT). To assess the extent of brain penetration, the ratio of unbound molecules (SN, LGZ and PCM) in brain / blood (the partition coefficient, Kp) was calculated as AUC_bECF_/AUC_blood_.

### Preparation of Drug Solutions

For preparation of SN injection solution in pharmacological studies, SN (CAS No. 6080-33-7, Catalog No. 110774, obtained from the National Institute for Food and Drug Control, Beijing, China, purity > 99%) was dissolved in DMSO (CAS No. 67-68-5, Catalog No. 34869, obtained from Sigma-Aldrich, Germany, purity > 99%), then mixed with Cremophor EL oil (CAS No. 61791-12-6, Catalog No. 238470, Sigma-Aldrich, Germany) and saline by vortex mixer (2,500 rpm for 1 min, Bibby Scientific, UK) using the volume rate of 1:4:5. Any further dilution was mixed with saline (CAS No. 7647-14-5, Catalog No. S0817, obtained from Sigma-Aldrich, Germany). Vehicle for SN was the above-mentioned dissolving solution. For preparation of SN injection solution in pharmacokinetic studies, SN (CAS No. 6080-33-7, Catalog No. 9582, obtained from Finetech Industry Limited, China, purity > 99%) was dissolved in saline. For preparation of LGZ injection solution in pharmacological and pharmacokinetic studies, LGZ (CAS No. 76494-51-4, Catalog No. 3628, obtained from Sinova Inc., USA, purity > 99%) was dissolved in saline. For preparation of PCM injection solution in pharmacological and pharmacokinetic studies, PCM (CAS No. 103-90-2, Catalog No. 1706, obtained from Tocris Cookson Ltd., UK, purity > 99%) was dissolved in saline.

### Statistics

All pharmacological experiments were conducted blindly. Data were presented as mean ± SEM, and were analyzed using analysis of variance (ANOVA) followed by Dunn's or Dunnett's multiple comparisons test, and / or Bonferroni's multiple comparisons test. *P* < 0.05 was considered as statistically significant.

## Results

### Analgesic Effects of SN, LGZ, or PCM on Mechanical and Heat Hypersensitivities in Rat Model of Incisional Pain

**Figure 1:**
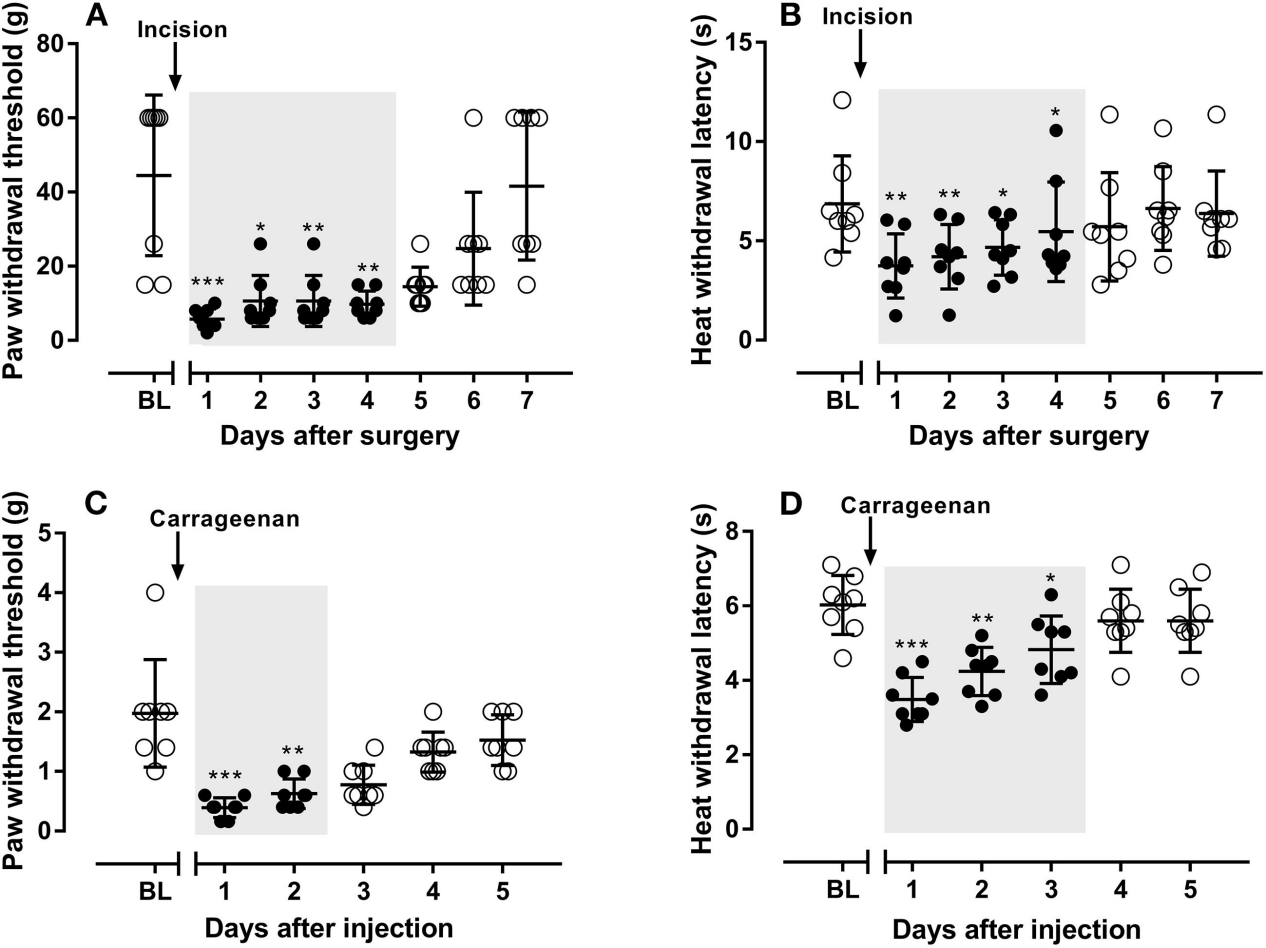
The development of mechanical **(A)** and heat **(B)** hypersensitivities in rats after incision in their hand paws, and the development of mechanical **(C)** and heat **(D)** hypersensitivities in mice after carrageenan injection in their hand paws. *N* = 8 animals, data were presented as mean ± SD. *P < 0.05, **P < 0.01, ***P < 0.001, mechanical **(A,C)** or heat thresholds **(B,D)** from Day 1 to 7 in rats **(A,B)** or from Day 1 to 5 in mice **(C,D)** were compared with their baseline values, using Dunn's **(A,C)** or Dunnett's multiple comparisons test **(B,D)** following ANOVA. Gray shaded areas represent durations that rats or mice experiencing ongoing hypersensitivities.

Twenty four hours after the incision in the plantar area in the hind paw, rats developed mechanical (Figure 1A) and heat (Figure 1B) hypersensitivities, which both lasted for 4 days. All pharmacology studies were done between 24 to 48 h after incision while animals exhibit maximum of developed mechanical and heat allodynia.

**Figure 2:**
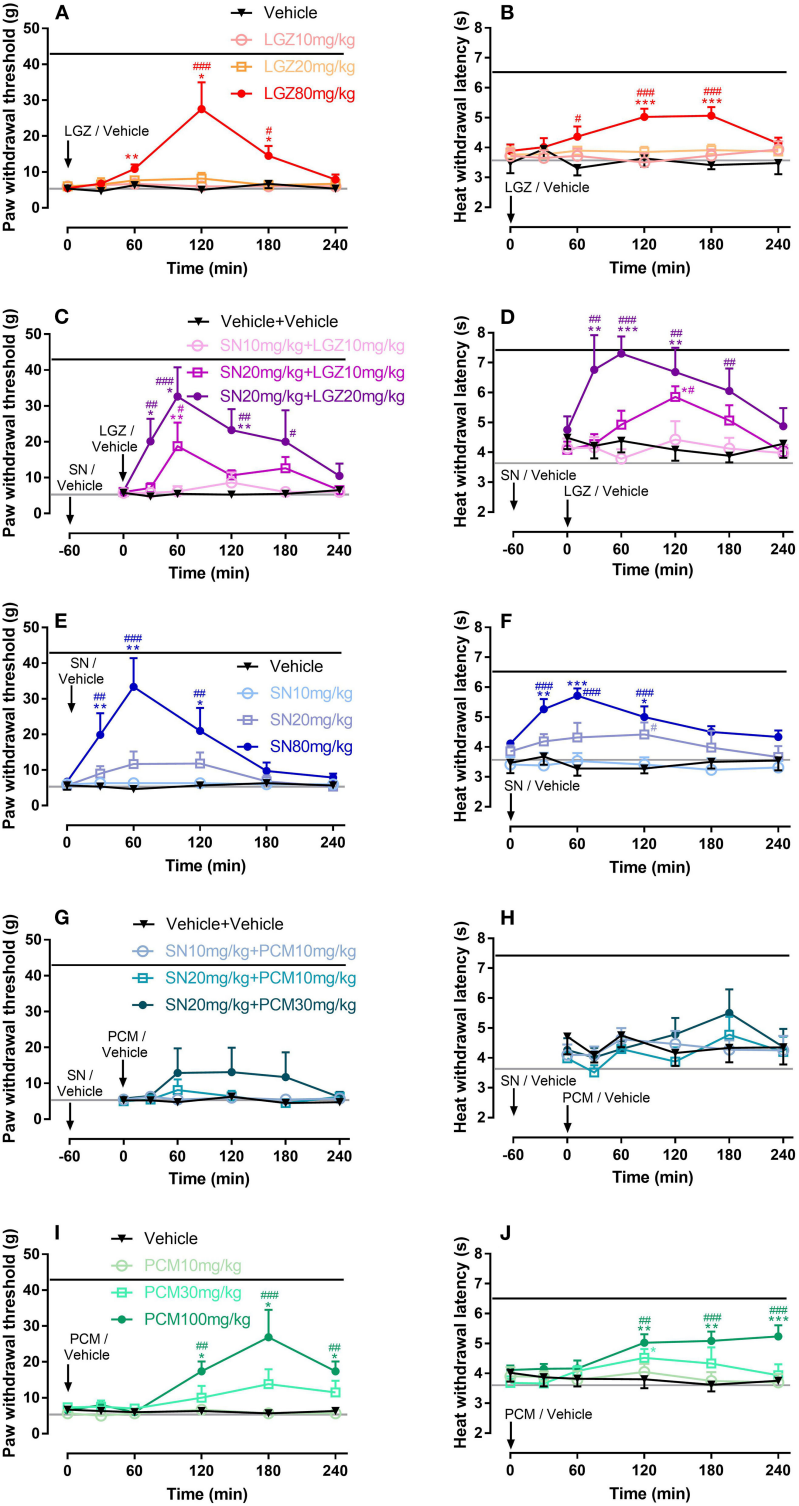
Dose dependent effects of LGZ **(A,B)**, SN **(E,F)**, PCM **(I,J)**; and SN combined with LGZ **(C,D)** or PCM **(G,H)** on mechanical and heat hypersensitivities in rats with incisional pain. *N* = 6–8 rats, data were presented as mean ± SEM. Black or Gray lines represent the average (mechanical / heat) thresholds, before or 1 day after incision, respectively. LGZ was applied at the dosages of 10, 20, and 80 mg/kg **(A,B)**; SN was applied at the dosages of 10, 20, and 80 mg/kg **(E,F)**; PCM was applied at the dosages 10, 30, and 100 mg/kg **(I,J)**; SN (at 10 or 20 mg/kg) were applied 60 min before LGZ (at 10 or 20 mg/kg; **C,D**), or PCM (at 10 or 30 mg/kg; **G,H**) administrations. Mechanical/heat thresholds were measured for 240 min after completion of drug applications. Two Way ANOVA with repeated measures indicated overall significant differences (*P* < 0.05) between the groups **(A–J)**. *P < 0.05, **P < 0.01, ***P < 0.001, post-drug thresholds were compared with pre-drug baselines at 0 min, using Dunn's **(A,C,E,G,I)** or Dunnett's **(B,D,F,H,J)** multiple comparisons test following ANOVA; ^#^*P* < 0.05, ^*##*^*P* < 0.01, ^*###*^*P* < 0.001, post-drug effects were compared with vehicle treatments at each time points, using Bonferroni's multiple comparisons test following ANOVA.

Single dosages of SN at 80 mg/kg (with analgesia lasted from 30 to 120 min, Figure 2E), LGZ at 80 mg/kg (with analgesia lasted from 60 to 180 min, Figure 2A), and PCM at 100 mg/kg (with analgesia lasted from 120 to 240 min, Figure 2I), but not SN at 10 and 20 mg/kg (Figure 2E), LGZ at 10 and 20 mg/kg (Figure 2A), or PCM at 10 and 30 mg/kg (Figure 2I) produced significant analgesic effects to mechanical stimuli. Similarly, heat hyperalgesia was significantly reversed by i.p. SN at 80 mg/kg (from 30 to 120 min, Figure 2F), LGZ at 80 mg/kg (from 60 to 180 min, Figure 2B), and PCM at 30 mg/kg (only at 120 min, Figure 2J) or 100 mg/kg (from 120 to 240 min, Figure 2F), but not by SN at 10 and 20 mg/kg (Figure 2F), LGZ at 10 and 20 mg/kg (Figure 2B), or PCM at 10 mg/kg (Figure 2J).

### Combinational Therapy of SN With LGZ or PCM in Rat Model of Incisional Pain

When SN was applied first and then LGZ administrated 60 min after, such combination produced significant analgesic effects for mechanical hypersensitivity from 30 to 180 min in “SN 20 mg/kg + LGZ 20 mg/kg” treatment, and at 60 min in “SN 20 mg/kg + LGZ 10 mg/kg” treatment (Figure 2C); Similarly, SN and LGZ combination also significantly alleviated heat hyperalgesia from 30 to 180 min in “SN 20 mg/kg + LGZ 20 mg/kg” treatment, and at 120 min in “SN 20 mg/kg + LGZ 10 mg/kg” treatment (Figure 2D). Even though, monotherapies of SN or LGZ at single dosages of 10 or 20 mg/kg failed to produce any analgesia (Figures 2A,B,E,F). Unlike SN plus LGZ, SN and PCM combinations (at sub-effective dosages) did not generate significant analgesic effect (Figures 2G,H).

When 240 min's analgesic effect was compared using Area Under Curve (AUC), “SN 20 mg/kg + LGZ 20 mg/kg” group was significantly greater than “SN 10 mg/kg” group, or “LGZ 10 and 20 mg/kg” groups (**Figures 4A,B**). In addition, mean of AUCs in group “SN 20 mg/kg + LGZ 20 mg/kg” was higher than that of “SN 80 mg/kg” and “LGZ 80 mg/kg” groups, representing a synergistic effect between SN and LGZ (combined at sub-effective dosages) in incisional pain model. Rats showed no signs of observable side effects including sedation, itching or severe allergy during pharmacological treatment periods.

### Analgesic Effects of SN, LGZ, or PCM on Mechanical and Heat Hypersensitivities in Mouse Model of Carrageenan Induced Inflammatory Pain

Similar as reported previously (Gao et al., 2013), 24 h after subcutaneous carrageenan injection (in plantar area of hind paws), mice developed marked mechanical (Figure 1C) and heat (Figure 1D) hypersensitivities, which lasted for 2 and 3 days, respectively. All pharmacology studies were performed between 24 to 48 h after carrageenan injection, while animals exhibit maximum allodynia.

**Figure 3:**
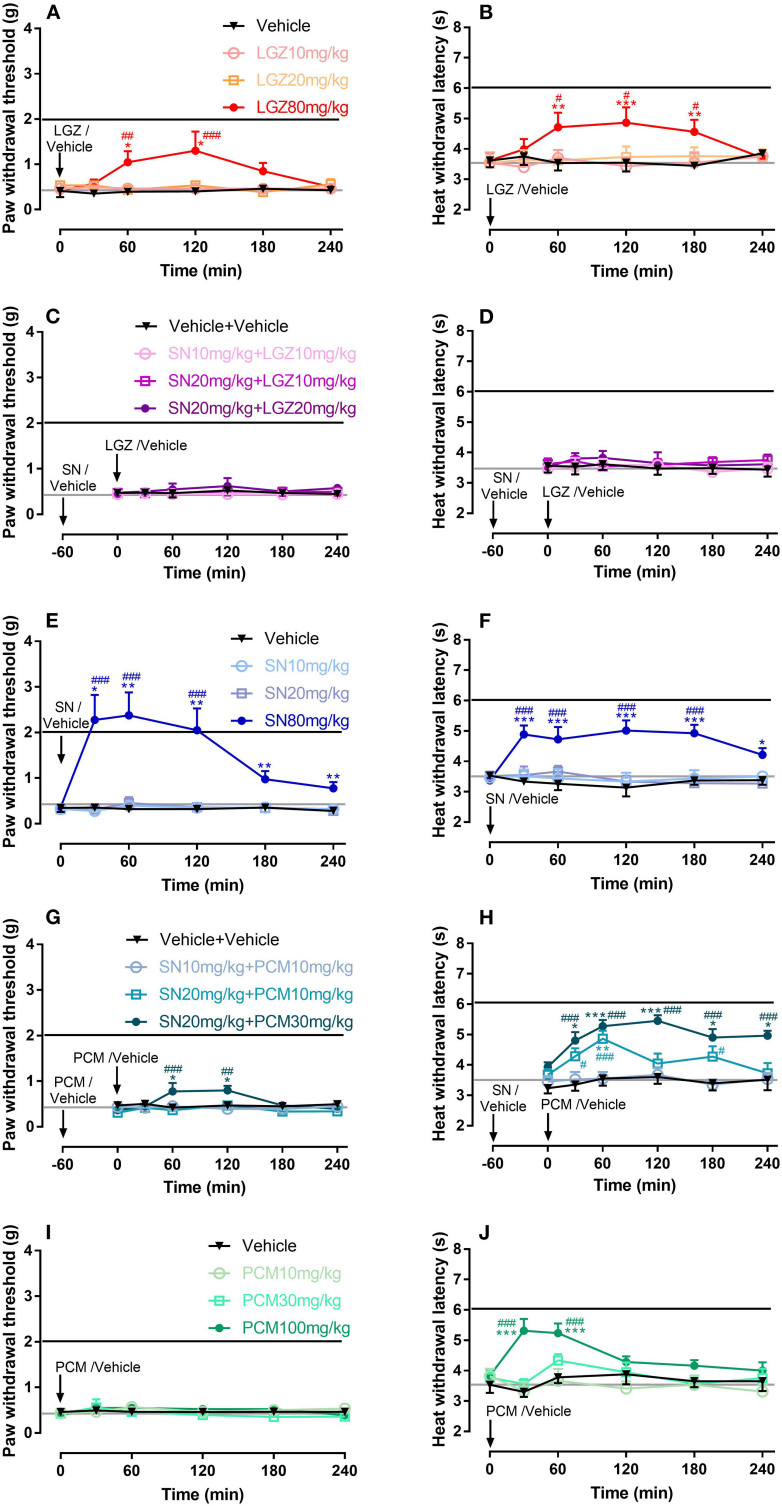
Dose dependent effects of LGZ **(A,B)**, SN **(E,F)**, PCM **(I,J)**; and SN combined with LGZ **(C,D)** or PCM **(G,H)** on mechanical and heat hypersensitivities in mice with carrageenan induced inflammatory pain. *N* = 6–8 mice, data were presented as mean ± SEM. Black or Gray lines represent the average (mechanical/heat) thresholds before or 1 day after carrageenan injection, respectively. LGZ was applied at the dosages of 10, 20, and 80 mg/kg **(A,B)**; SN was applied at the dosages of 10, 20, and 80 mg/kg **(E,F)**; PCM was applied at the dosages 10, 30, and 100 mg/kg **(I,J)**; SN (at 10 or 20 mg/kg) were applied 60 min before LGZ (at 10 or 20 mg/kg; **C,D**), or PCM (at 10 or 30 mg/kg; **G,H**) administrations. Mechanical/heat thresholds were measured for 240 min after completion of drug applications. Two Way ANOVA with repeated measures indicated overall significant differences (*P* < 0.05) between the groups **(A,B,E,F,G,H,J)**. *P < 0.05, **P < 0.01, ***P < 0.001, post-drug thresholds were compared with pre-drug baselines at 0 min, using Dunn's **(A,C,E,G,I)** or Dunnett's **(B,D,F,H,J)** multiple comparisons test following ANOVA; ^#^*P* < 0.05, ^*##*^*P* < 0.01, ^*###*^*P* < 0.001, post-drug effects were compared with controls (vehicle treatments) at each time points, using Bonferroni's multiple comparisons test following ANOVA.

Single dosages of SN at 80 mg/kg (with analgesia lasted from 30 to 240 min, Figure 3E), and LGZ at 80 mg/kg (with analgesia lasted from 60 to 120 min, Figure 3A), but not SN at 10 and 20 mg/kg (Figure 3E), LGZ at 10 and 20 mg/kg (Figure 3A), or PCM at 10, 30, and 100 mg/kg (Figure 3I) produced significant analgesic effects to mechanical stimuli. Similarly, heat hyperalgesia was significantly reversed by SN at 80 mg/kg (from 30 to 240 min, Figure 3F), LGZ at 80 mg/kg (from 60 to 180 min, Figure 3B), and PCM at 100 mg/kg (from 30 to 60 min, Figure 3J), but not by SN at 10 or 20 mg/kg (Figure 3F), LGZ at 10 or 20 mg/kg (Figure 3B), and PCM at 10 or 30 mg/kg (Figure 3J).

### Combinational Therapy of SN With LGZ or PCM in Mouse Model of Carrageenan Induced Inflammatory Pain

When SN was pretreated for 60 min and then combined with LGZ, no significant analgesic effects was produced by “SN 10 mg/kg + LGZ 10 mg/kg”, “SN 20 mg/kg + LGZ 10 mg/kg” or “SN 20 mg/kg + LGZ 20 mg/kg” groups (Figures 3C,D). However, “SN 20 mg/kg + PCM 30 mg/kg” treatment generated significant anti-hyperalgesic effects against both mechanical (from 60 to 120 min, Figure 3G) and heat (from 30 to 240 min, Figure 3H) hypersensitivities. In addition, “SN 20 mg/kg + PCM 10 mg/kg” treatment also significantly alleviated heat allodynia (at 30, 60, and 180 min, Figure 3H).

**Figure 4:**
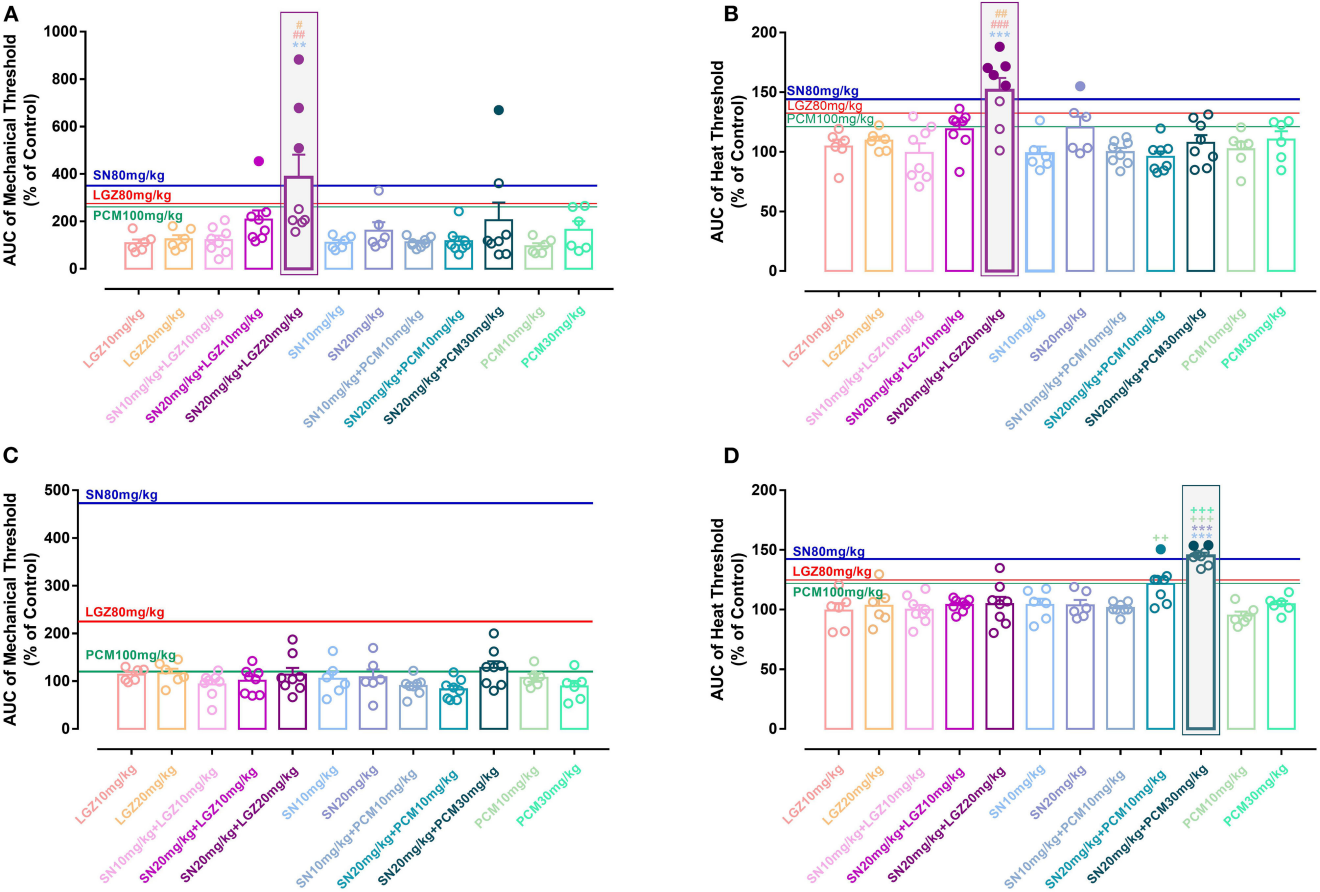
Area Under Curve (AUC) normalized with control (vehicle animals) for the effects of single or combined therapies of SN, LGZ, and PCM at different dosages against mechanical and heat hypersensitivities in rats with incisional pain **(A,B)**, or in mice with carrageenan induced inflammatory pain **(C,D)**. *N* = 6–8 animals, data were presented as mean ± SEM. Blue, red, or green lines represent the average AUCs of mechanical/heat thresholds for SN 80 mg/kg, LGZ 80 mg/kg, or PCM 100 mg/kg treatment, respectively. Filled circles indicating AUCs of drug combinations were larger than the average AUCs of effective single drug treatments (SN 80 mg/kg and LGZ 80 mg/kg). AUCs of drug combinations were compared with SN (at 10 or 20 mg/kg, **P < 0.01, ***P < 0.001, significant differences with specific groups were illustrated by respective representing colors), LGZ (at 10 or 20 mg/kg, ^#^*P* < 0.05, ^*##*^*P* < 0.01, ^*###*^*P* < 0.001, significant differences with specific groups were illustrated by respective representing colors); or PCM (at 10 or 30 mg/kg, ++*P* < 0.01, +++*P* < 0.001, illustrated by respective representing colors), using Bonferroni's multiple comparisons test following ANOVA. Gray shaded area marked the significant (and most effective) dosage combination for the combined drug therapies.

When 240 min's analgesic effect was compared using AUC, “SN 20 mg/kg + PCM 30 mg/kg” group was significantly greater than “SN 10 and 20 mg/kg” group, or “PCM 10 and 30 mg/kg” groups, in heat (Figure 4D) but not in mechanical (Figure 4C) hypersensitivities, showing drug's efficacy is dissociated between two pain modalities. In addition, for heat thresholds, mean of AUC for group “SN 20 mg/kg + PCM 30 mg/kg” group was higher than that of “SN 80 mg/kg” and “PCM 100 mg/kg” groups, representing a synergistic effect between SN and PCM (at sub-effective dosages) in carrageenan induced inflammatory pain model (Figure 4D). Mice showed no signs of observable side effects including sedation, itching or severe allergy during drug treatment periods.

### Effects of LGZ or PCM on the Pharmacokinetic Parameters of SN in Combinational Formulas

**Figure 5:**
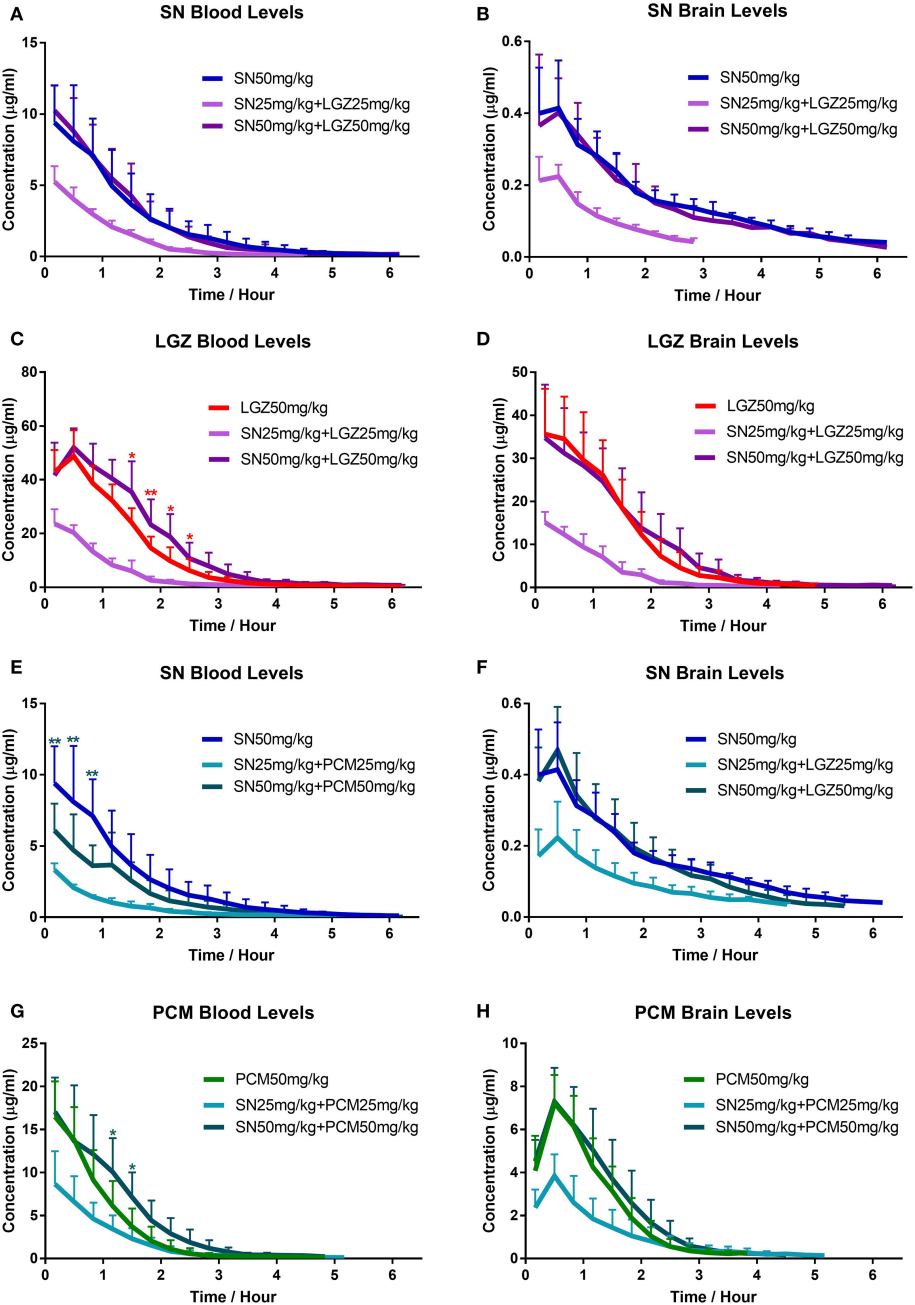
Investigation of pharmacokinetics changes of SN, LGZ, and PCM in combined formulas. Drug concentrations of SN, LGZ, and PCM in blood **(A,C,E,G)** and striatum of the brain **(B,D,F,H)** were monitored continuously in collected samples (using microdialysis), after intravenous injection of SN, LGZ, and PCM at 50 mg/kg or SN combine with LGZ/PCM at 50 or 25 mg/kg, in rats. *N* = 6 rats for each group. Data was presented as mean ± SD. Two Way ANOVA indicated significant differences between the groups **(A–H)**. *P < 0.05, **P < 0.01, drug concentrations of different groups were compared at each time points using Bonferroni's multiple comparisons test following ANOVA (significant differences with specific groups were illustrated by respective representing colors).

After intravenous injection of SN in rats, levels of SN in brain followed same tendency of that in the blood with certain delay (Figures 5A,B). Tmax in brain was 0.33 h. Cmax in brain was 0.43 μg·mL^−1^ (equivalent to 4.3% of the Cmax in blood, which was 10.07 μg·mL^−1^; Table 1). For t_1/2_, there was no significant difference between brain and blood. However, mean retention time (MRT) in brain extracellular fluid was higher than that in blood (1.75 h > 1.12 h, *P* = 0.008; Table 1). In terms of the brain permeability of SN, Kp value is from 0.06 to 0.07 (Table 1), indicating poor SN penetration from blood to brain. When combined with LGZ (SN 50 mg/kg + LGZ 50 mg/kg), the parameters (Cmax, MRT, t_1/2_, and AUC) of SN (at 50 mg/kg), in blood and brain extracellular fluids remained stable. Even in low dose SN and LGZ combination (SN 25 mg/kg + LGZ 25 mg/kg), the parameters (MRT, t_1/2_ clearance, and Kp value) of SN remained stable. These facts indicating LGZ does not influence the pharmacokinetic properties of SN in the blood and in extracellular fluids of brain, when combined.

**Table 1:** Effects of LGZ or PCM on the pharmacokinetic parameters of SN in blood and extracellular fluid of rat brain tissue.

**PK parameter**	**SN levels**				
	**SN**	**SN + LGZ**	**SN + LGZ**	**SN + PCM**	**SN + PCM**
	**(50 mg·kg^**−1**^)**	**(50 mg·kg^**−1**^ + 50 mg·kg^**−1**^)**	**(25 mg·kg^**−1**^ + 25 mg·kg^**−1**^)**	**(50 mg·kg^**−1**^ + 50 mg·kg^**−1**^)**	**(25 mg·kg^**−1**^ + 25 mg·kg^**−1**^)**
**BLOOD**					
AUC_0−*t*_ (h·μg·mL^−1^)	14.80 ± 6.13	15.19 ± 4.45	6.25 ± 1.02^**^	7.72 ± 3.02^*^	3.72 ± 0.58^**^
AUC_0−∞_ (h·μg·mL^−1^)	15.01 ± 6.20	15.42 ± 4.50	6.36 ± 1.04^**^	7.84 ± 3.07^*^	3.84 ± 0.6^**^
MRT (h)	1.12 ± 0.28	1.05 ± 0.16	0.87 ± 0.08	1.13 ± 0.14	0.98 ± 0.24
t1/2 (h)	0.94 ± 0.26	0.97 ± 0.30	0.80 ± 0.17	0.87 ± 0.21	0.96 ± 0.21
Vz (L·kg^−1^)	5.30 ± 2.45	4.64 ± 1.31	4.56 ± 0.94	7.60 ± 2.95	9.01 ± 1.49^**^
CLz (L·h^−1^·kg^−1^)	3.75 ± 1.31	3.46 ± 0.90	4.01 ± 0.59	6.35 ± 2.61^*^	6.65 ± 1.11^**^
Cmax (μg·mL^−1^)	10.07 ± 2.75	10.38 ± 1.60	5.26 ± 1.09^**^	6.11 ± 1.91^*^	3.32 ± 0.46^**^
**BRAIN**					
AUC_0−*t*_ (h·μg·mL^−1^)	0.91 ± 0.17^##^	0.77 ± 0.25^##^	0.32 ± 0.04^**^^##^	0.89 ± 0.29^##^	0.41 ± 0.13^**^^##^
AUC_0−∞_ (h·μg·mL^−1^)	0.99 ± 0.15^##^	0.89 ± 0.26^##^	0.39 ± 0.05^**^^##^	0.93 ± 0.32^##^	0.48 ± 0.13^**^^##^
MRT (h)	1.75 ± 0.37^##^	1.42 ± 0.39	1.05 ± 0.05^**^^##^	1.60 ± 0.08^##^	1.47 ± 0.39^#^
t1/2 (h)	1.14 ± 0.27	1.22 ± 0.19	1.18 ± 0.34	1.09 ± 0.34	1.35 ± 0.34
Tmax (h)	0.33 ± 0.18	0.33 ± 0.18	0.39 ± 0.17	0.50 ± 0.00	0.37 ± 0.18
Cmax (μg·mL^−1^)	0.43 ± 0.13^##^	0.45 ± 0.14^##^	0.24 ± 0.05^**^^##^	0.47 ± 0.12^##^	0.23 ± 0.09*^##^
**BRAIN-TO-BLOOD AUC RATIOS**					
Kp	0.07 ± 0.03	0.06 ± 0.02	0.06 ± 0.01	0.12 ± 0.01^**^	0.11 ± 0.03^**^

*N = 6 rats, data presented as Mean ± SD*.

* *P < 0.05*,

** P < 0.01, Parameters of SN in SN+LGZ or SN+PCM groups were compared with those in the SN group, respectively;

# *P < 0.05*,

## *P < 0.01, parameters of extracellular fluid of rat brain tissue were compared with that of blood tissue, respectively*.

In SN and PCM combination at 50 mg/kg (Figures 5E,F), t_1/2_ of SN in the extracellular fluids of brain or blood was not significantly altered; In blood (Table 1), SN's clearance increased significantly (*P* < 0.05), while Cmax and AUC (14.80 μg·mL-1→9.28 μg·mL^−1^) decreased significantly (*P* < 0.05), correlated with respective changes in concentration curves (Figure 5E) and AUCs (Supplementary Figure 4E); However, SN's Cmax and AUC in extracellular fluids of brain remained steady. In addition, SN's Kp value was increased (0.07→0.12, *P* < 0.01; Table 1).

### Effects of SN on the Pharmacokinetic Parameters of LGZ or PCM in Combinational Formulas

After intravenous injection, LGZ appeared rapidly in blood (Figure 5C) and extracellular fluids of the brain (Figure 5D). LGZ concentration reached peak in brain at the period of 0–20 min (Figure 5D). Cmax in brain (39.16 μg·mL^−1^) was about 78% of the Cmax in blood (50.04 μg·mL^−1^; *P* < 0.05; Table 2). MRT and t_1/2_ remained steady in brain extracellular fluids and in blood. Kp value for LGZ was from 0.60 to 0.69 (Table 2), representing good blood-brain permeability. After combined with SN (at 50 mg/kg), parameters of LGZ (Cmax, clearance, t1/2, MRT, AUC and Kp) in blood and brain were not significantly altered, showing that SN does not affect the pharmacokinetics of LGZ in the blood and brain when combined.

**Table 2:** Effects of SN on the pharmacokinetic parameters of LGZ or PCM in blood and extracellular fluid of rat brain tissue.

**PK parameter**	**LGZ levels**			**PCM levels**		
	**LGZ**	**LGZ + SN**	**LGZ + SN**	**PCM**	**PCM + SN**	**PCM + SN**
	**(50 mg·kg^**−1**^)**	**(50 mg·kg^**−1**^ + 50 mg·kg^**−1**^)**	**(25 mg·kg^**−1**^ + 25 mg·kg^**−1**^)**	**(50 mg·kg^**−1**^)**	**(50 mg·kg^**−1**^ + 50 mg·kg^**−1**^)**	**(25 mg·kg^**−1**^ + 25 mg·kg^**−1**^)**
**BLOOD**						
AUC_0−*t*_ (h·μg·mL^−1^)	76.73 ± 14.27	94.94 ± 18.75	26.87 ± 5.83^**^	18.06 ± 5.94	23.78 ± 9.08	9.96 ± 3.7^*^
AUC_0−∞_ (h·μg·mL^−1^)	78.02 ± 14.06	96.24 ± 19.02	27.9 ± 5.89^**^	18.2 ± 6.01	24.08 ± 9.18	10.08 ± 3.71^*^
MRT (h)	1.04 ± 0.11	1.21 ± 0.22	0.78 ± 0.14^**^	0.70 ± 0.17	0.92 ± 0.16^*^	0.87 ± 0.37
t1/2 (h)	1.36 ± 0.30	1.40 ± 0.27	1.29 ± 0.43	0.73 ± 0.22	0.85 ± 0.31	0.77 ± 0.32
Vz (L·kg^−1^)	1.48 ± 0.58	1.07 ± 0.27	1.71 ± 0.56	2.91 ± 0.28	2.84 ± 1.38	3.05 ± 1.70
CLz (L·h^−1^·kg^−1^)	0.66 ± 0.13	0.54 ± 0.10	0.93 ± 0.20^*^	3.03 ± 1.07	2.37 ± 0.98	2.81 ± 1.09
Cmax (μg·mL^−1^)	50.04 ± 8.83	51.99 ± 7.08	23.63 ± 5.37^**^	16.53 ± 4.1	17.12 ± 4.08	8.66 ± 3.82^**^
**BRAIN**						
AUC_0−*t*_ (h·μg·mL^−1^)	53.81 ± 17.72^#^	57.81 ± 22.38^#^	16.38 ± 4.25^**^^##^	9.3 ± 2.22^##^	10.81 ± 3.90^##^	5.01 ± 2.14^#^
AUC_0−∞_ (h·μg·mL^−1^)	54.57 ± 18.11^#^	58.42 ± 22.6^#^	16.82 ± 4.18^**^^##^	9.43 ± 2.27^##^	10.95 ± 3.96^##^	5.08 ± 2.17^#^
MRT (h)	1.03 ± 0.12	1.18 ± 0.17	0.85 ± 0.06^*^	0.99 ± 0.14^##^	1.09 ± 0.14	1.09 ± 0.35
t1/2 (h)	1.06 ± 0.13	1.19 ± 0.43	1.19 ± 0.36	0.50 ± 0.11	0.59 ± 0.07	0.58 ± 0.19
Tmax (h)	0.28 ± 0.17	0.17 ± 0.00	0.17 ± 0.00	0.56 ± 0.14	0.50 ± 0.00	0.50 ± 0.00
Cmax (μg·mL^−1^)	38.14 ± 9.21^#^	34.73 ± 12.37^#^	15.1 ± 2.45^**^^##^	7.24 ± 1.31^##^	7.29 ± 1.57^##^	3.85 ± 1.00^**^^#^
**BRAIN-TO-BLOOD AUC RATIOS**						
Kp	0.69 ± 0.16	0.60 ± 0.17	0.62 ± 0.11	0.54 ± 0.14	0.47 ± 0.1	0.54 ± 0.28

*N = 6 rats, data presented as Mean ± SD*.

* *P < 0.05*,

** P < 0.01, Parameters of LGZ/PCM in SN+LGZ or SN+PCM groups were compared with those in the LGZ/PCM group, respectively;

# *P < 0.05*,

## *P < 0.01, parameters of extracellular fluid of rat brain tissue were compared with those of blood tissue, respectively*.

After intravenous injection of PCM, the levels of PCM in brain followed the same propensity of that in the blood with certain delay (Figures 5G,H). In brain (Table 2), Tmax was about 0.56 h, and Cmax was 7.24 μg·mL^−1^, which was about 44% of the Cmax in blood (16.53 μg·mL^−1^). No significant change for PCM's t_1/2_ was observed in blood or brain. The Kp value for PCM is from 0.47 to 0.54 (Table 2), revealing PCM has a moderate the blood-brain permeability. After the combination with SN (at 50 mg/kg), the parameters of PCM (Cmax, MRT, t1/2, AUC, and Kp) in blood and brain were not significantly changed. Albeit, a tendency of increased blood PCM concentrations in the “SN 50 mg/kg + PCM 50 mg/kg” group (compared with PCM 50 mg/kg group) could be seen (from the respective concentration curves, Figure 5G; and AUCs, Supplementary Figure 4G).

## Discussion

Current analgesics against postoperative or inflammatory pain are restricted by incomplete efficacy, and some potent agents such as opioids are limited by side effects (Chou et al., 2016; Häuser and Fitzcharles, 2017). Synergistic interactions of drug combinations might provide superior analgesia with fewer adverse outcomes than monotherapy by targeting of multiple mechanisms (Gilron et al., 2013). In such regard, we used SN in combinational formulas and demonstrated that therapies of SN plus LGZ or SN plus PCM in sub-effective concentrations, produced synergistic analgesia, while avoided opioid-related adverse effects. Indeed, the observed synergism is expected and could be explained by different mechanisms of anti-hyperalgesic actions. Intriguingly, however, we discovered that the synergistic interaction was subjected to the pain models and differed between mechanical and heat modalities. Pharmacokinetics of SN in blood was influenced by coadministration of PCM but not LGZ, which could partially explain the pharmacological discoveries. Topics related to the study results are discussed below.

### Synergistic Effects of SN Plus LGZ on Incisional Pain and SN Plus PCM on Carrageenan Induced Heat Hyperalgesia

A pathological change in the nociceptive system induced by incision or inflammation could generate persistent alterations in nociceptors that drive hypersensitivity. SN plus LGZ or PCM might restore the nociceptor to its normal state by their synergistic anti-hyperalgesic mechanisms.

In incisional pain condition, the development of primary hyperalgesia was found to be mediated by sensitizations of primary afferents and dorsal horn neurons (Brennan et al., 2005), and activation of NMDA receptors (Woolf and Thompson, 1991). The analgesic effect of the NMDA receptor antagonist ketamine, can be enhanced by Ligustrazine (Liu et al., 2018). Taking consideration that resembling dextromethorphan (Yamasaki, 1976), SN may also work as a weak NMDA antagonist (Qian et al., 2007, Gao et al., 2007), it is possible that synergy between LGZ and SN on incisional pain is dependent on NMDA receptor system inhibition.

In inflammatory pain condition, changes in the chemical milieu induces excessive prostaglandins, that sensitize the nociceptors (Woolf and Ma, 2007). PCM is generally considered to be a weak inhibitor of prostaglandins (Graham and Scott, 2005). In like manner, SN can systemically suppress the generation of prostaglandins, both *in vitro* and *in vivo* (Liu et al., 1994; Qian et al., 2007). Therefore, it is possible that synergism of SN plus PCM arises from mutual inhibition on the prostaglandin synthesis. Besides, SN also showed capability of reducing various key inflammatory mediators other than prostaglandins (Liu et al., 1994; Wang et al., 2005; Chacur et al., 2010). While the antinociceptive effect of paracetamol was thought to be dependent on spinal serotonergic systems (Tjølsen et al., 1991). Thus, synergy between SN and PCM (in inflammatory pain), might also be achieved based on their complementary mechanisms.

### The Inversed Result When Comparing SN Plus PCM vs. SN Plus LGZ Between the Incisional and the Inflammatory Model

In the present study synergy between SN and LGZ was lost in carrageenan induced inflammatory pain condition, while synergy between SN and PCM was lost in incisional pain condition. This inversed result indicates nociceptive system was differentially affected by SN combinations in two pain scenarios.

Surgical damage to peripheral nerve fibers is a key element for incisional pain. However, acute inflammatory pain is mainly mediated through the local inflammatory mediators, and has less “central component.” Studies have shown that both LGZ (Gao et al., 2008; Wang et al., 2016) and SN (Gao et al., 2013, 2014), but not PCM, were effective against pain following various types of nerve injuries. In addition, we have seen a pronounced “central effect,” such as inhibition of the increased spontaneous neuronal activation, and suppression of the enlarged peripheral receptive field of dorsal horn neurons (Coderre et al., 1993), being produced by SN plus LGZ against chronic pain (Gao et al., 2019a). These facts support the rationale why SN combined with LGZ but not PCM could generate synergistic effect on incisional pain. On the other hand, in inflammatory pain conditions, it is possible that SN and LGZ combination could not sufficiently “silence” the peripheral nociceptors sensitized by the “inflammatory soup,” as the SN and PCM combination did.

In addition, the differing effects of SN combinations on carrageenan induced heat hyperalgesia could be partially linked to the pharmacokinetics changes induced by drug interaction, that SN and PCM when combinedly used, might result in a reduction of SN blood concentration and a probable increase in PCM concentration. As SN showed broad analgesic characteristics especially on incisional pain and neuropathic pain (Gao et al., 2013; Zhu et al., 2016), while PCM has an analgesic profile more reserved to inflammatory pain. It is reasonable to postulate that the pharmacokinetic change induced by SN and PCM combination, drives a shift in the analgesic potency toward a better cure for inflammatory pain (than incisional pain).

### Dissociation of Mechanical and Heat Pain Modalities in Response to PCM or SN Plus PCM on in Carrageenan Induced Inflammatory Pain Condition

Hypersensitivities to mechanical and heat stimuli are predominately mediated by A-fiber and C-fiber nociceptors. In primate, A-fiber nociceptors are divided into type I A mechano-heat (AMH) units and type II AMH units (Djouhri and Lawson, 2004). Type I AMHs are noxious mechano-sensors, which predominately comprised of Aδ fibers with a few moderate pressure receptors (Aβ fibers). Type II AMHs are noxious heat-sensors, comprised of thinly myelinated Aδ fibers (Djouhri and Lawson, 2004; Woolf and Ma, 2007). Unmyelinated C-fiber nociceptors do not appear to play a major role in mechanical pain, but are heat specific nociceptors. The detection of heat and mechanical pain modalities are also mediated by specific ion channels and receptors which are localized in mature nociceptors (Woolf and Ma, 2007). For instance, heat sensitivity is mediated by multiple TRP channels—TRPV1, TRPV2, TRPV3, and TRPV4 (Dhaka et al., 2006). Looked from their threshold, TRPV1 (activated by temperature >42°C) and TRPV2 (activated by temperature >52°C) overlapped with heat pain (Caterina et al., 1999). On the other hand, mechanical sensitivity is recently revealed to be depending on activation of Piezo channels (Coste et al., 2012).

Interestingly, in mice with carrageenan induced inflammatory pain, we found PCM monotherapy reduced heat but not mechanical hypersensitivity. In addition, SN and PCM combination also only worked on heat hyperalgesia. Thus, a dissociation between mechanical and heat pain modalities has been seen in response to PCM analgesics. An explanation of this dissociation is the activities of Type I AMHs (noxious mechano-sensors) that accounting for the establishment of mechanical allodynia after inflammation might be not sufficiently suppressed by PCM analgesics. While on the hand, Type II AMHs (noxious heat-sensors) and C fibers with the heat responsive receptors TRPV1 and TRPV2 (Caterina et al., 1999), which are “sleeping nociceptors” that becoming “wake” in the presence of inflammation (Woolf and Ma, 2007), could be more sensitive to PCM analgesics. Cellular and molecular mechanisms accounting for this dissociation of drug's effects are still await elucidation. However, such phenomenon is not unique. The dissociations between different pain modalities could be also found in acute nociceptive pain or incisional pain models under physiological modulation of pain associated receptors (Pogatzki-Zahn et al., 2005; Kayser et al., 2007).

### Pharmacokinetic Changes Induced by SN and PCM Combination

SN could induce a concentration dependent lowering effect on the transepithelial electrical resistance of Caco-2 cell monolayers, which was completely reversible (Lu et al., 2010), suggesting SN may altering the membrane transportation in blood brain barrier (increase the transportation of co-administered compounds). On contrary, PCM may reduce the transportation of co-administered molecules such as imatinib (Nassar et al., 2009). It is reasonable to expect that SN plus LGZ or PCM could generate drug-drug interactions that modulate the exposure of each drug in blood and brain. In this study, we were able to reveal that, when SN was combined with LGZ, pharmacokinetic properties of each drug were not altered. However, when SN was combined with PCM, the blood SN concentrations were decreased and there was a tendency of increase in blood PCM concentrations. The reduction of SN blood levels by coadministration of PCM indicates that PCM (and its metabolites) possibly increased expression of transporter proteins (Slitt et al., 2003), especially p-glycoprotein which actively excretes SN (Tsai and Wu, 2003). PCM might also enhance SN's elimination pathway from the kidney by glomerular filtration/active tubular secretion, or facilitate SN's metabolism thorough activating specific metabolic enzymes such as cytochrome P450, though further investigation is still needed to confirm the exact mechanism.

### Safety Concerns for SN Based Drug Combinations

At lethal dose (oral around 1,000 mg/kg in rats), SN can produce apparent sedation, which was soon exacerbated by an increased reflex excitability or seizure, followed by general muscular weakness and reduction in respiration, leading to fatal asphyxia (Yamasaki, 1976). In previous study, when SN was applied at the dosages larger than 80 mg/kg (oral or i.p.), only minor sedation in locomotion (without any sign of respiratory inhibition) could be seen (Gao et al., 2013). In the present study, we have not observed any sign of side effects in SN applied at 10, 20, and 80 mg/kg or in combinational formulas with LGZ or PCM. Taking consideration that repeated administration of SN did not generate tolerance, but increased the baseline thresholds in animals with chronic pain (Gao et al., 2014), the effective combinations used in this study could be safely used for long-terms.

Systemic opioids are widely used pre-/post-surgery (Chou et al., 2016), even at the risk of inducing tolerance/addiction, and eliciting delayed hyperalgesia and allodynia (Revat et al., 2002; Pergolizzi et al., 2011). We have proved that the analgesic efficacies of SN (including SN plus LGZ) could not be blocked by the opioid receptor antagonist Naloxone (Gao et al., 2015, 2019a). Therefore, combination of SN and LGZ could be useful in clinical settings as an opioid alternative medicine for controlling postoperative pain.

## Conclusions

Efforts to develop novel analgesics that surpass the limitation of current treatments have not been successful. Therefore, combination therapy remains an important beneficial strategy. In the current study, we have demonstrated that at sub-effective doses, combined therapy of SN with LGZ significantly reduced mechanical and heat hypersensitivities induced by incision, and combined therapy of SN with PCM was effective against carrageenan induced heat hyperalgesia, without generating observable side effects. The pharmacological discoveries could be partially explained by pharmacokinetic alterations in SN combinations. Although further clinical validation is still needed, these observed synergies advocate the potential benefits of using SN plus LGZ for postoperative pain management, and using SN plus PCM for controlling inflammatory pain.

## Data Availability Statement

HPLC-MS/MS data were uploaded to FigShare; doi: 10.6084/m9.figshare.11545581.

## Ethics Statement

The animal study was reviewed and approved by Stockholm Ethical Committee, Research Ethics Committee at China Academy of Chinese Medical Sciences.

## Author Contributions

JG, YH, DW, J-DJ, and TG initiated the study and decided the research goal. TG, TL, DW, and JG have major contributions for accomplishing research study. JG, TG, TL, and DW analyzed research data. All authors were involved in establishing the research protocol, preparing the manuscript, and approved the final version of the manuscript.

## Conflict of Interest

WJ and WF were employed by the company Zhejiang Zhenyuan Pharmaceutical Co., Ltd. The remaining authors declare that the research was conducted in the absence of any commercial or financial relationships that could be construed as a potential conflict of interest.
