# Supplementary material for: Antinociceptive Effects of Sinomenine Combined With Ligustrazine or Paracetamol in Animal Models of Incisional and Inflammatory Pain
Source: Front Physiol. 2021 Feb 9;11:523769. doi: 10.3389/fphys.2020.523769 (PMC7900506; doi:10.3389/fphys.2020.523769)
Supplement: Supplementary file 1 [file Data_Sheet_1.docx]

**SUPPLEMENTARY MATERIAL**


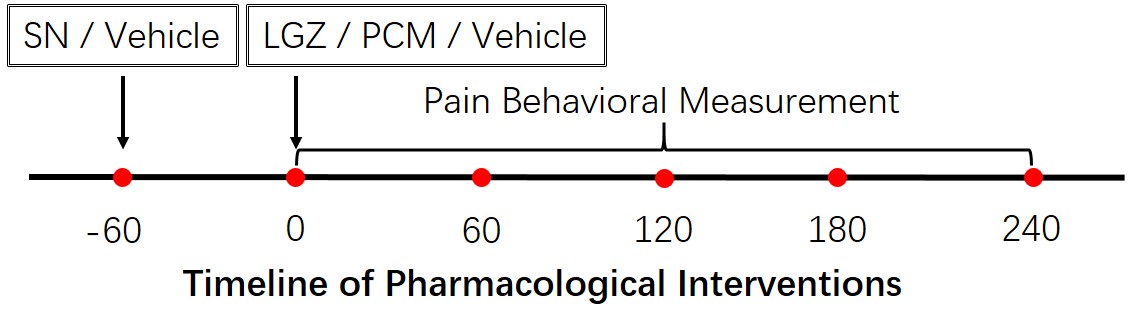


**Figure S1**

Caption of Fig.S1. Time line of pharmacological interventions. SN / Vehicle was given 60 min prior to the application of LGZ / PCM / Vehicle, to insure the optimal analgesic efficacy. Pain behavioral measurement (measurement of mechanical allodynia and heat hyperalgesia) was performed after application of LGZ / PCM / Vehicle, for 240 min.


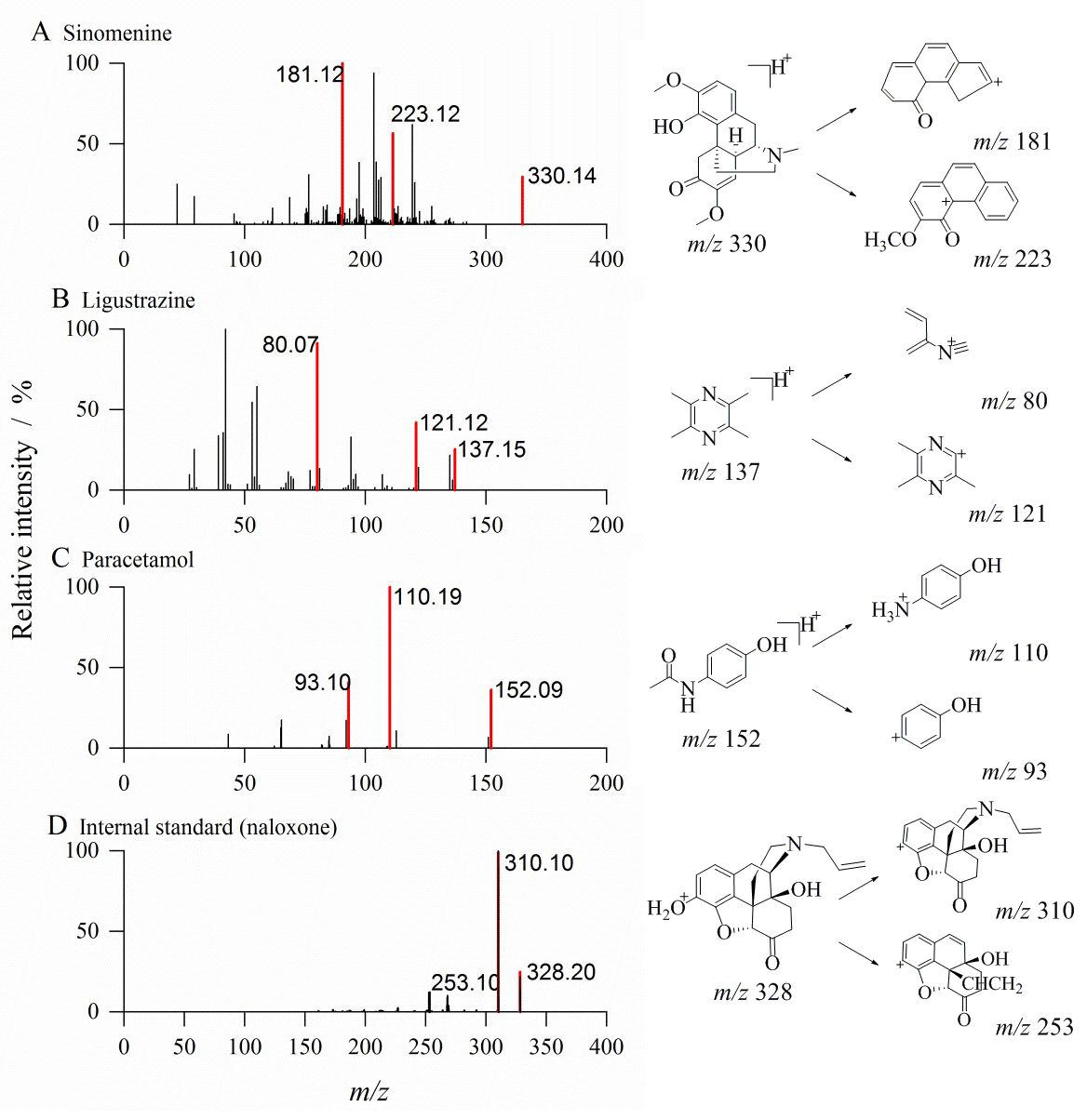


**Figure S2**

Caption of Fig.S2. Product ion spectrums and fragmentation pattern of SN, LGZ, PCM and naloxone (internal standard). The precursor ion and the major product ions are marked with red thick bars in the figure. All spectrums have been normalized to the largest peak.


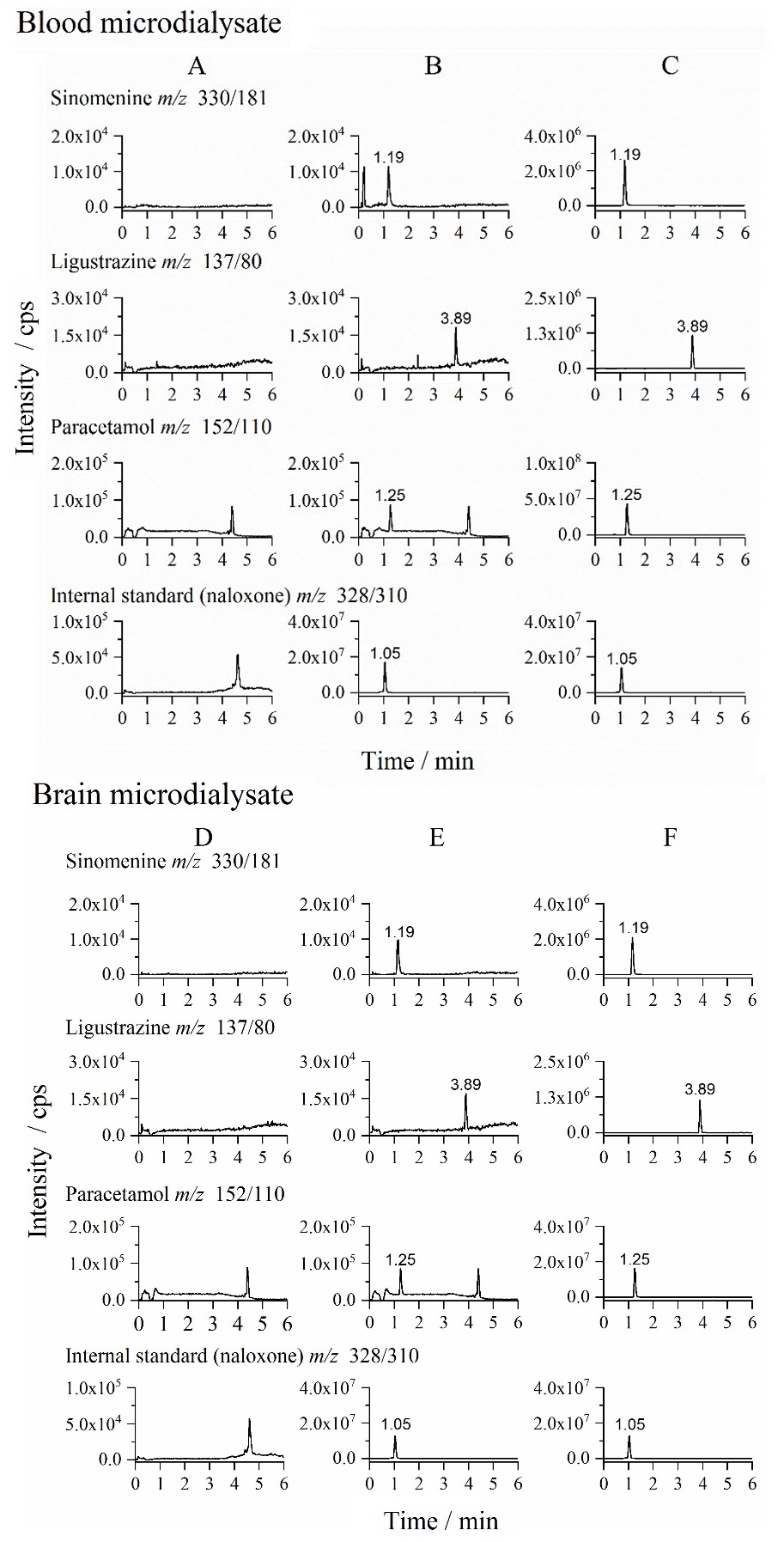


**Figure S3**

Caption of Fig.S3. Representative extraction ion chromatograms of blank rat blood (A) and brain (D) microdialysate sample; blank blood (B) and brain (E) microdialysate with internal standard (naloxone); and the blood (C) and brain (F) microdialysate samples collected from 0-20 min after the intravenous administration of sinomenine (50 mg·kg^−1^), ligustrazine (50 mg·kg^−1^), and paracetamol (50 mg·kg^−1^) to rats. The quantifier multiple-reaction monitoring transition and retention time is indicated and shown for each compound.


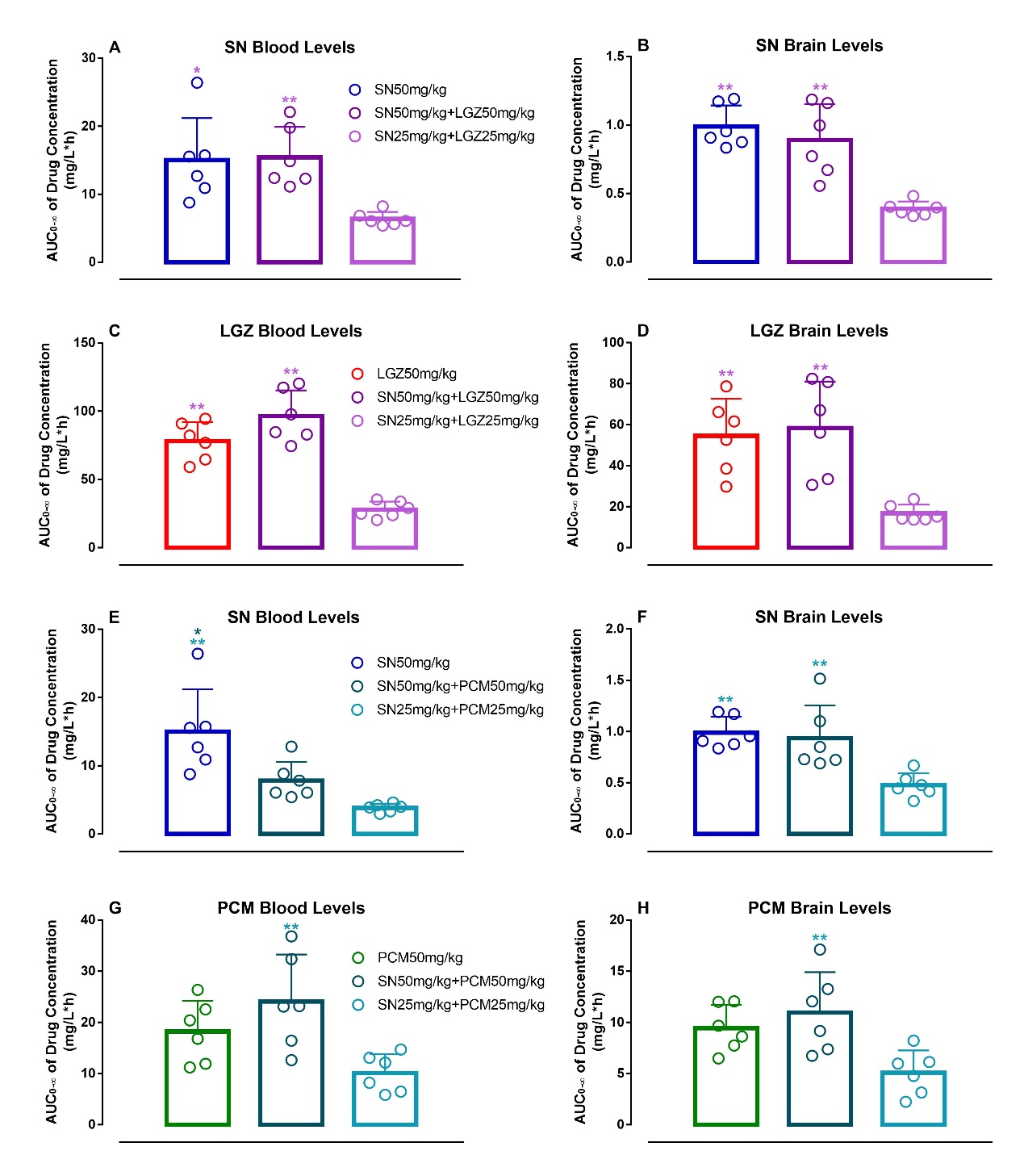


**Figure S4**

Caption of Fig.S4. Area Under Curve (AUC) 0–∞, of the drug concentrations of SN，LGZ, and PCM in blood (A, C，E, G) and in striatum of the brain (B, D, F, H) in rats, after intravenous injection of SN, LGZ and PCM at 50mg/kg, or SN combine with LGZ / PCM at 50mg/kg or 25mg/kg. N=6 rats for each group. Data was presented as mean ± SD. ANOVA indicated significant differences between the groups (A, B, C, D, E, F, G, H). *P<0.05，**P<0.01, AUCs of drug concentrations in different groups were compared with each other at each time points using Bonferroni's multiple comparisons test following ANOVA (significant differences with specific groups were illustrated by respective representing colors).

| Compound | MRM Fragments | Dwell Time (ms) | Declustering Potential (V) | Collision Energy (eV) | Rentiom Time (min) |
| --- | --- | --- | --- | --- | --- |
| Sinomenine | 330/181 | 80 | 158 | 44 | 1.19 |
| Ligustrazine | 137/80 | 80 | 74 | 41 | 3.89 |
| Paracetamol | 152/110 | 80 | 30 | 24 | 1.25 |
| Naloxone (Internal standard) | 328/310 | 80 | 50 | 28 | 1.05 |

**Table S1**

Caption of Table S1. Ions and fragmentations used in multiple-reaction monitoring (MRM) mode for each compound.
